# High-Performance Infrared Photodetectors Based on Graphene Nanoribbon Vertical Heterojunctions via Dissociated Double-Walled Carbon Nanotubes

**DOI:** 10.3390/nano16100625

**Published:** 2026-05-19

**Authors:** Ziheng Li, Yu Sun, Muyang Li, Nan Han, Zeyuan Wang, Jihui Fan, Hui Zhou, Xiaoqing Jiang, Jie Li, Yafei Ning, Klaus Leifer, Mingyang Wang, Ming Gao, Hu Li, Aimin Song

**Affiliations:** 1 Shandong Key Laboratory of Next-Generation Semiconductor Technology and Systems, School of Integrated Circuits, Shandong University, Jinan 250101, China; 202332338@mail.sdu.edu.cn (Z.L.); 202212334@mail.sdu.edu.cn (H.Z.);; 2 Shandong Hi-Speed Information Group Co., Ltd., Jinan 250102, China; 3 Shenzhen Research Institute, Shandong University, Shenzhen 518063, China; 4 Department of Engineering Sciences, Uppsala University, 75121 Uppsala, Sweden; 5 State Key Laboratory of Electronic Thin Films and Integrated Devices, Institute of Fundamental and Frontier Sciences, University of Electronic Science and Technology of China, Chengdu 611731, China; 6 Academy of Intelligent Innovation, Shandong University, Jinan 250101, China; 7 Institute of Nanoscience and Applications, Southern University of Science and Technology, Shenzhen 518055, China; a.song@manchester.ac.uk; 8 Department of Electrical and Electronic Engineering, University of Manchester, Manchester M139PL, UK

**Keywords:** double-walled carbon nanotubes, graphene nanoribbons, vertical heterojunctions, near-infrared photodetectors

## Abstract

Graphene nanoribbons (GNRs) inherit the exceptional carrier mobility of graphene while offering tunable bandgaps, making them promising for high-performance optoelectronics. Here, we report a high-performance near-infrared photodetector based on a p-GNR/Al_2_O_3_/n-Si vertical heterojunction, where GNR is directly produced by dissociating double-walled carbon nanotubes (DWCNTs). The 10 nm Al_2_O_3_ interlayer serves as an effective barrier and passivation layer, suppressing dark current and enhancing interfacial charge separation. Under 1064 nm illumination, the device delivers outstanding performance. At −6 V bias, the responsivity and detectivity reach 159.55 A/W and 2.01 × 10^12^ Jones, respectively. Notably, under zero-bias self-powered mode, it still achieves a high responsivity of 8.71 A/W, a detectivity of 1.15 × 10^13^ Jones, and a fast response time of 307.5 μs. These results fully validate the feasibility of GNR-based heterojunctions for high-performance optoelectronic devices and pave the way for their future integration into low-power, high-sensitivity photodetection systems and next-generation optoelectronic integrated circuits.

## 1. Introduction

Photodetectors are devices that convert optical signals into electrical signals and they are widely used in military, industrial, and civilian fields such as military detection, optical communication, aerospace, and biochemical analysis [1,2,3,4,5,6,7,8,9]. Infrared photodetectors convert invisible infrared radiation into measurable electrical signals, which are divided into two categories: thermal detection and photon detection. Photon detectors have received widespread attention due to their excellent detection performance. However, traditional photon-based infrared photodetectors rely on low-temperature cooling systems, which hinder miniaturization and portability [10,11,12,13,14]. Meanwhile, most traditional photodetectors require external bias voltage to separate photo-generated electron–hole pairs, resulting in increased device size, high energy consumption, and limited response speed caused by depletion-region interference [15,16,17]. Therefore, the development of high-performance infrared photodetectors with room temperature, miniaturization, and self-powered has become an urgent research focus to meet the needs of future compact and low-power nanodevices [3,18,19,20].

The performance of photodetectors is mainly determined by the semiconductor active layer and device structure. Traditional narrow bandgap semiconductors (such as silicon) are widely used in the manufacturing of infrared photodetectors due to their abundant reserves and mature processing techniques [21,22,23], but their low carrier mobility (<1000 cm^2^V^−1^s^−1^) limits the response frequency and computational speed [24]. Two-dimensional (2D) materials have inherent advantages such as high carrier mobility, adjustable bandgap, and suspended bondless surfaces, and have become promising candidates for room temperature shortwave infrared photodetectors [25,26]. The van der Waals heterojunction formed by 2D materials exhibits a strong built-in electric field at the interface, which can effectively separate photo-generated carriers and suppress dark current [27,28]. In 2D materials, graphene has excellent optical and electrical properties, but there are issues with its zero bandgap and lack of gain mechanism, resulting in high dark current and low responsivity [29]. Graphene nanoribbons (GNRs) are derived from graphene with a nanoscale width, inheriting high carrier mobility while obtaining adjustable bandgaps and spin-polarized edge states through quantum confinement effects, effectively addressing the limitations of graphene [30,31,32,33]. In addition, vertical heterojunctions are superior to planar structures in terms of integration density, carrier transport speed, and device miniaturization, making them an ideal choice for the next generation of optoelectronic devices [34].

In order to achieve high-performance infrared photodetectors, this work fabricated p-GNR/Al_2_O_3_/n-Si vertical heterojunction shortwave infrared photodetectors. The GNR layer was derived from double-walled carbon nanotubes (DWCNTs). Considering that interface quality significantly affects heterojunction performance, a thin film of 10 nm aluminum oxide (Al_2_O_3_) is inserted between the Si substrate and GNR as a barrier and passivation layer to enhance the interface barrier, passivate defects, and suppress dark current [35,36]. The photoelectric performance test shows that the responsivity of the device under −6 V bias is 159.55 A/W and the detectivity is 2.01 × 10^12^ Jones. It is worth noting that in self-powered mode (0 V bias), it still maintains a high responsivity of 8.71 A/W and a detectivity of 1.15 × 10^13^ Jones. This study provides a feasible solution for high-performance and low-cost self-powered infrared photodetectors, with broad application prospects.

## 2. Materials and Methods

### 2.1. Preparation of GNR

A total of 25 mg of DWCNTs (Ossila, M2016L2, diameter: 2–4 nm, length: 48 μm, Sheffield, UK) were first heated in air at 300 °C for 30 min. Next, 25 mL of 98% concentrated sulfuric acid (H_2_SO_4_, Yantai Yuandong Fine Chemicals Co., Ltd., Yantai, China) was added, followed by stirring at room temperature for 2 h. Then 75 mg of potassium permanganate (KMnO_4_, Sigma Aldrich, St. Louis, MO, USA) was introduced into the mixture, which was subsequently heated to 45 °C and stirred for 3.5 h. After the reaction, 200 mL of pre-chilled deionized water was added. Once the exothermic process subsided, the mixture was filtered, washed repeatedly, and air-dried at room temperature. For dispersion, 20 mg of the dried product was added to 200 mL of a pre-configured 1% sodium dodecyl benzene sulfonate (SDBS, Sigma Aldrich, St. Louis, MO, USA) aqueous solution, followed by 1 h of ultrasonic treatment to achieve homogeneous dispersion. The resulting GNR suspension was purified by vacuum filtration, with the GNR deposited on the filter membrane. After drying, the GNR-deposited portion of the membrane was cut out and preserved for subsequent device fabrication.

This solution-based GNR preparation strategy relies on two core steps: first, strong oxidants including concentrated sulfuric acid and potassium permanganate are used to introduce edge defects on DWCNTs, then ultrasonic treatment is applied to split the DWCNTs along these defect sites and form GNRs. Distinct from conventional GNR exfoliation methods, this approach precisely controls the oxidant dosage to ensure a relatively low oxidation degree in the final product. A critical factor for successful DWCNT dissociation is the effective dispersion of nanotubes in the liquid phase, as the hydrophobic surface properties of DWCNTs tend to cause aggregation and impede the reaction process. To address this issue, SDBS was selected as the surfactant in this work. its hydrophobic alkyl chains can adsorb onto the DWCNT surface, while the hydrophilic groups extend outward to improve the water dispersibility of nanotubes and facilitate their separation from aggregates. Notably, residual SDBS may adversely affect the performance of GNR-based photodetectors by altering the conductivity and surface roughness of GNR films, as well as introducing carrier scattering centers that reduce carrier lifetime and mobility, thereby decreasing device responsivity. To mitigate such effects, the GNR films were subjected to multiple vacuum filtration washes before deposition. Furthermore, annealing the films at 300 °C in an inert gas atmosphere for 2 h was performed to thoroughly remove residual SDBS [37].

### 2.2. Preparation of Photodetectors

**Figure 1:**
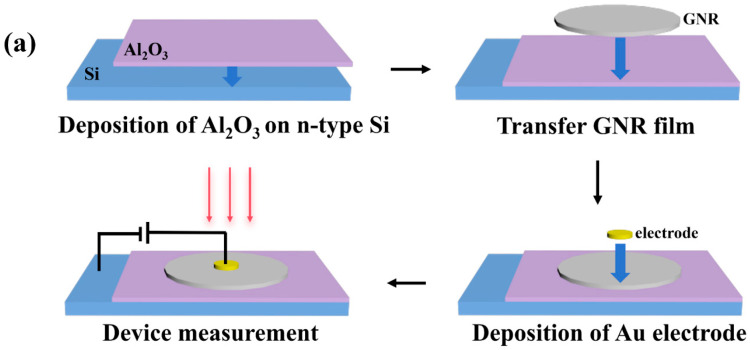

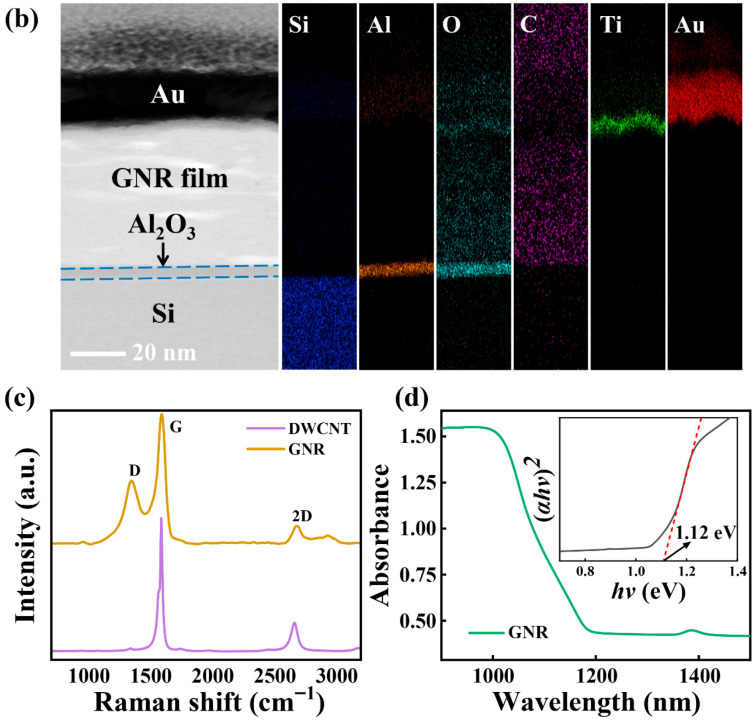
(**a**) Schematic diagram of the manufacturing process of the GNR/Al_2_O_3_/Si photodetector. (**b**) TEM cross-sectional and mapping image of the GNR/Al_2_O_3_/Si photodetector. (**c**) Raman spectra of a GNR and DWCNT. (**d**) The absorption spectra of a GNR (the illustration shows the Tauc plot of a GNR with an optical bandgap of 1.12 eV).

Figure 1a shows a schematic diagram of the manufacturing process of a GNR/Al_2_O_3_/Si photodetector. The n-type silicon wafer (SiBranch Microelectronics, Ningbo, China) was cut to a size of about 1.2 cm × 1.2 cm, and 10 nm Al_2_O_3_ was deposited on top using an atomic layer-deposition device. Place the silicon wafer with the deposited Al_2_O_3_ and the dried filter membrane with the deposited GNRs into a Petri dish containing an appropriate amount of acetone. After the filter membrane is completely dissolved by acetone, the GNR membrane is effectively separated. Use the silicon wafer to remove the GNR film and obtain GNR/Al_2_O_3_/Si. After drying in air, anneal for 2 h under inert gas and heat at 300°C. Ti/Au electrodes (thickness 10 nm/50 nm, actual area 0.1 mm^2^) were then grown on the GNRs using an electron-beam evaporation method to obtain a photodetector with GNR/Al_2_O_3_/Si structure.

## 3. Results

### 3.1. Characterization and Testing

Obtain Raman spectra using a RENISHAW Invia instrument and a 532 nm laser (Wotton-under-Edge, UK). The cross-section of the photodetector was characterized using a FEI Talos F200X G2 transmission electron microscope (TEM, Hillsboro, OR, USA). The absorption and transmission spectra of the GNRs were tested using a Shimadzu UV-3600i Plus UV visible near-infrared spectrophotometer (Kyoto, Japan). The I-V and I-T characteristics of the photodetector were measured using Agilent 2902A (Santa Clara, CA, USA). In order to evaluate the optoelectronic performance, the device was tested under a laser with a wavelength of 1064 nm. The laser power density was measured using an Ophir Nova II (Jerusalem, Israel) optical power density meter, and the response time was measured using a Keysight InfiniiVision MSO6004A oscilloscope (Santa Clara, CA, USA).

### 3.2. Results and Discussion

The significant photo-response of the GNR/Al_2_O_3_/Si photodetector is primarily attributed to the light absorption capability of the GNR layer. A GNR film with insufficient thickness will impair its light-harvesting efficiency, thereby reducing the device’s responsivity. Conversely, an excessively thick GNR film may lead to an increased residual content of conductive metallic CNT, which in turn elevates the dark current. Thus, the preparation of GNR films with an appropriate thickness is crucial for optimizing device performance. Optical microscopy images can provide preliminary insights into the morphology of thin films and the structural integrity of devices. Figure S1 displays the optical micrograph of the photodetector, from which it can be observed that the GNR film exhibits a non-compact surface with numerous pores. Following transfer and annealing, the GNR film was examined by atomic force microscopy (AFM). Figure S2 displays the surface morphology, thickness characterization, and three-dimensional topography. These results confirm that the film is uniform and consistent. Figure 1b presents the TEM cross-sectional and mapping image of the device, which reveals the elemental distribution across different layers of the GNR/Al_2_O_3_/Si heterojunction and confirms the successful implementation of the proposed fabrication process. The distinct contrast between bright and dark regions in the TEM image corresponds to the different functional layers of the photodetector. Figure 1c,d demonstrates the successful preparation of GNR using different characterization methods. Figure 1c shows the Raman spectra of GNR and pristine DWCNT on a silicon substrate. Both GNR and DWCNT exhibit a G peak around 1590 cm^−1^, which is caused by the stretching vibration of equivalent C-C bonds and is a characteristic peak of CNT. Both exhibit 2D peaks around 2680 cm^−1^, which are caused by non-equivalent C-C bonds and reflect the number of layers in graphene. However, only GNR exhibits a D peak at 1350 cm^−1^, which is generated by topological edge defects and structural defects in GNR, synergistically driving dual-resonance Raman scattering. This indicates that the defects are caused by the dissociation of the DWCNTs, confirming successful preparation through mild chemical dissociation. In the GNR samples, the G peak at 1590 cm^−1^ and the 2D peak at 2680 cm^−1^ are characteristic in-plane vibrations of the sp^2^ atoms in graphene, which are sensitive to the number of graphene layers. The ratio of 2D to G peak intensity (I2D/IG) is an identifier for single-layer graphene, where values close to or higher than 2 typically indicate a high-quality and defect-free sample. It is worth noting that in the DWCNT and GNR samples, the intensity of the G peak always exceeds that of the 2D peak. The I2D/IG of the DWCNTs and GNRs are 0.22 and 0.14, respectively, indicating that they are multi-layer, and the decrease in the values is caused by the aggregation and overlap of the dissociated GNR [37]. These defects generated by dissociation inevitably alter the local carbon bonding states, resulting in a mixed configuration of sp2 and sp3 hybridization coexisting. Research has clearly confirmed that the carbon bonding state serves as a key factor in modulating the interfacial charge transfer, in which sp^2^-hybridized carbon actively participates in interfacial binding with metal oxides and effectively promotes the separation of photo-generated carriers [38]. In the GNR/Al_2_O_3_/Si photodetector presented in this work, such mixed bonding states can introduce trap states and trigger trap-assisted carrier multiplication mechanisms, which are responsible for the excellent optoelectronic performance in the photodetector. Figure 1d shows the absorption spectra obtained, indicating the occurrence of bandgap caused by the quantum confinement effect in the GNR. The optical bandgap of the GNR has been determined to be 1.12 eV based on calculations using the Tauc-plot method [39]. The Planck–Einstein relation illustrates the relationship between photon energy and wavelength:

(1) $$E\;=\;\frac{\mathrm{hc}}{\lambda }$$

Among them, *E*, c, h, and *λ* represent the photon energy, speed of light in vacuum, Planck constant, and photon wavelength, respectively. The relationship between the bandgap and absorption threshold can also be calculated using this formula:

(2) $$E_{g}\;=\;\frac{\mathrm{hc}}{\lambda_{g}}$$

Among them, *E_g_* and *λ_g_* represent the bandgap and absorption threshold, respectively. Input the bandgap obtained from the Tauc plot into Formula (1) to calculate the absorption threshold *λ_g_* = 1107 nm, which is basically compatible with the 1064 nm laser. Therefore, in this research, we use the 1064 nm laser to test the performance of the photodetector [40]. These analysis results not only verify the successful preparation of the GNRs, but also provide important references for the subsequent design and performance optimization of optoelectronic devices.

**Figure 2:**
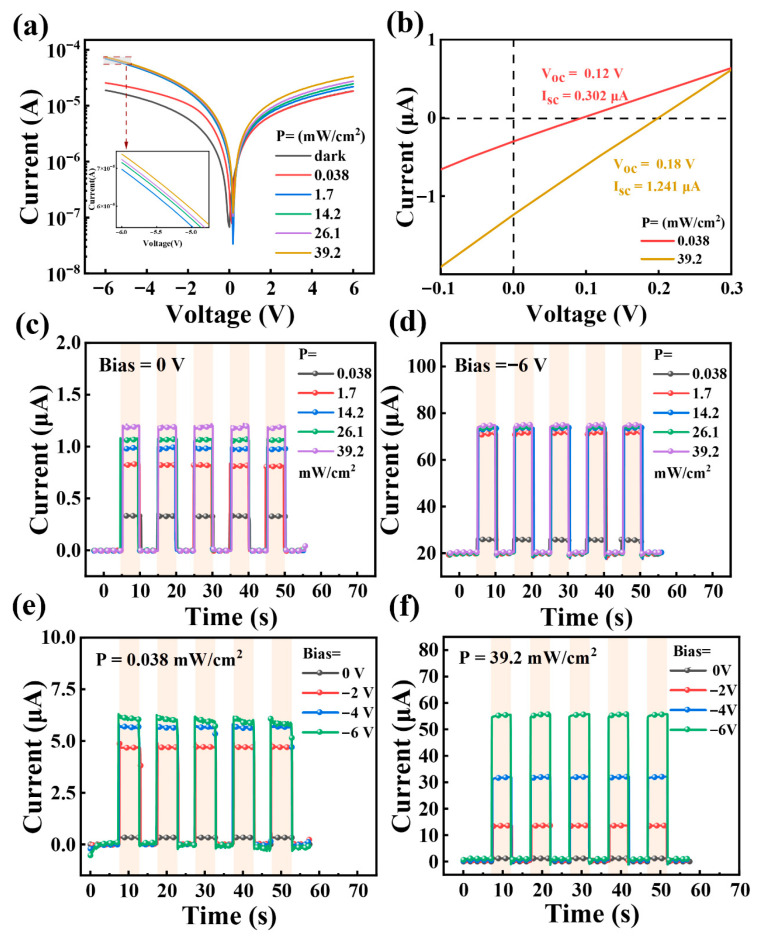
(**a**) Relationship between photocurrent and bias voltage of GNR/Al_2_O_3_/Si photodetector at different optical power densities. (**b**) The open-circuit voltage and short-circuit current vary with the change in light power density. Dynamic current response of photodetectors at different optical power densities with bias voltages of (**c**) 0 V and (**d**) −6 V. Dynamic current response of photodetectors at different bias voltages with optical power densities of (**e**) 0.038 mW/cm^2^ and (**f**) 39.2 mW/cm^2^.

Optical power density plays an important role in the performance evaluation of photodetectors. Therefore, the optical power density-dependent photo-response characteristics of heterojunction photodetectors were studied. The static (I-V curve) and dynamic response (I-T curve) characteristics of vertically stacked heterojunction devices were measured under 1064 nm laser irradiation in the dark and at different light powers. The light power density increased from 0.038 mW/cm^2^ to 39.2 mW/cm^2^, and the applied voltage varied from −6 to +6 V. Compared with the magnitude of dark current, the small photocurrent generated by photo-generated carriers under laser irradiation can have a negligible impact on the overall device. The time response curve is rectangular and shows no significant attenuation during multiple alternating bright and dark periods, indicating that the device has consistent repeatability. The reverse current and forward current under 1064 nm laser irradiation are significantly higher than those under dark conditions, exhibiting enhanced reverse bias conduction. The presence of Al_2_O_3_ suppresses dark current by designing interface barriers and passivation defects, effectively reducing the leakage pathways of charge carriers. We studied the effects of Al_2_O_3_, without Al_2_O_3_ and Al_2_O_3_ with different thicknesses (5 nm, 10 nm, and 15 nm) on the light response characteristics of the device. As shown in Figure 2 and Figures S3–S5, the results showed that Al_2_O_3_ with a thickness of 10 nm had the best performance and was selected as the optimal thickness. The excellent performance of 10 nm thick Al_2_O_3_ can be attributed to its reduction of direct contact between GNR and Si, minimizing unwanted electron tunneling and enhancing the transport of photoexcited charge carriers, thereby improving the sensitivity of the photodetector. In addition, Al_2_O_3_ provides an optimized path for carrier transport, which can block unnecessary current paths without excessively hindering carrier transport, thereby optimizing the photoelectric conversion efficiency [41].

As shown in Figure 2a, under dark conditions, the reverse saturation current of the GNR/Si heterojunction with a 10 nm Al_2_O_3_ interface layer is 18.81 μA. Due to the effective passivation of the Al_2_O_3_ thin layer, the generation of depletion-region holes and electrons under reverse bias is reduced, which is smaller than the value of the GNR/Si heterojunction without the Al_2_O_3_ thin layer (39.53 μA). The current of the photodetector under illumination is greater than that in the dark, exhibiting obvious photo-response characteristics. In addition, when the photodetector is irradiated with 1064 nm infrared light, the I-V curve shifts upward to the right relative to the curve under dark conditions, indicating that the device generates 0 V bias photocurrent [42]. Under 0.038 mW/cm^2^ laser irradiation, the photocurrent of the device increased from 0.30 μA to 25.52 μA, as the bias voltage changed from 0 V to −6 V. Under 39.2 mW/cm^2^ laser irradiation, the photocurrent of the device increased from 1.24 μA to 74.16 μA under the same bias voltage change. This relationship can also be understood in the subsequent analysis of Figure 2c,d [43]. As shown in Figure 2b, when the optical power density is 0.038 mW/cm^2^, the open-circuit voltage (V_OC_) and short-circuit current (I_SC_) are 0.12 V and 0.302 μA, respectively. When the optical power density increases to 39.2 mW/cm^2^, the V_OC_ and I_SC_ also increase to 0.18 V and 1.241 μA, respectively. It can be concluded that as the optical power density increases, both the V_OC_ and I_SC_ increase to a certain extent, and the V_OC_ gradually reaches saturation, indicating that the device has a self-powered effect. Figure 2c,d depict the real-time optical response of 1064 nm light with different bias voltages. From Figure 2c, the optical response of the photodetector at different optical power densities can be obtained when the bias voltage is 0 V. A photocurrent of 1.19 μA was observed at an optical power density of 39.2 mW/cm^2^, and the magnitude of the photocurrent during this period was positively correlated with the incident optical power at 0 V bias voltage, demonstrating its potential as an excellent self-powered photodetector. Figure 2d shows the I-T curve of the device at different optical power densities under −6 V bias. Compared with the photocurrent under 0 V bias, the photocurrent at all optical power densities is greatly enhanced under −6 V bias, indicating that the photocurrent can be adjusted by applying bias. Note that as the optical power density increases the photocurrent also monotonically increases to approximate saturation, as higher intensity illumination improves the quantum efficiency of the device until the photo-generated carriers saturate [44]. The separation process of photo-generated electron–hole pairs under laser irradiation is affected by the applied reverse bias voltage. Figure 2e,f further characterizes the time-dependent light response of the heterojunction under periodic-switching near-infrared illumination. The device is highly sensitive to incident light and can reversibly switch between high and low conductivity states, thereby generating stable and reproducible photocurrent. Figure 2e shows the I-T curve of GNR/Al_2_O_3_/Si photodetectors measured at optical power densities of 0.038 mW/cm^2^ under bias voltages of 0, −2, −4, and −6 V. When the laser is continuously turned on and off, there is a significant change in photocurrent, and a large amount of photocurrent is generated when turned on. The rising and falling edges are very steep, indicating that photoexcited electrons and holes can be rapidly generated and separated in heterojunctions. The photocurrent increases with the increase in reverse bias voltage and exhibits fast response time at all bias voltages. Figure 2f shows the I-T curve at a light power density of 39.2 mW/cm^2^ under bias voltages of 0, −2, −4, and −6 V. Under laser irradiation at 39.2 mW/cm^2^, as the bias voltage changes from 0 V to −6 V, the photocurrent increases from 1.24 μA to 74.16 μA. Figure 2e,f both indicate that as the reverse bias voltage increases, the photocurrent also gradually increases. This is because the support of bias voltage improves the efficiency of charge extraction [42].

For self-powered photodetectors, the built-in electric field is a key factor in separating photo-generated carriers without external bias. After depositing Al_2_O_3_ on the n-type silicon substrate and forming a PN heterojunction device with p-type GNR, a built-in electric field from silicon to GNR will be formed in the depletion region due to carrier diffusion when reaching thermal equilibrium. When the PN heterojunction is illuminated by infrared light, photo-generated charge carriers will be generated inside the device, and the built-in electric field promotes the separation of electron–hole pairs, transferring photo-generated electrons from GNR to Si. At the same time, a large number of photo-generated holes migrate and accumulate in the GNR valence band, resulting in reverse photocurrent under 0 V bias. Electrons and holes are collected by the negative and positive electrodes respectively and then recombined through an external circuit to ultimately achieve the self-powering function of the photodetector [42].

The definition of the photo–dark current ratio (PDCR) is

(3) $$\mathrm{PDCR}=\frac{\left|J_{p}-J_{d}\right|}{\left|J_{d}\right|}$$

**Figure 3:**
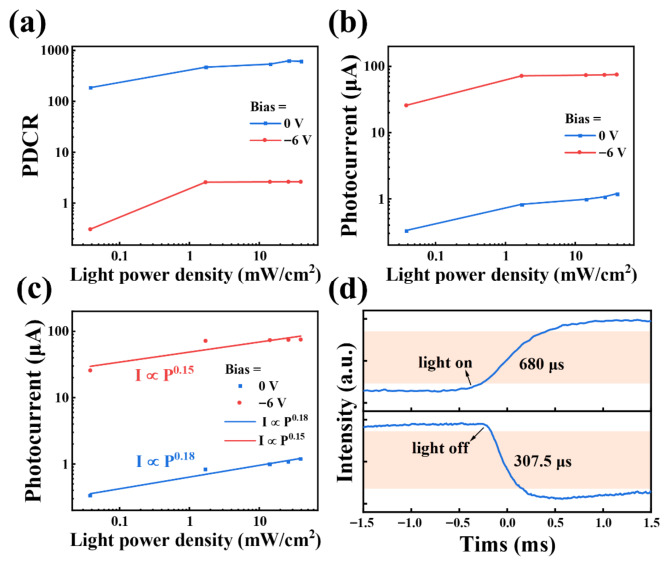
(**a**) Changes in PDCR with optical power density under 0 V and −6 V bias voltages. (**b**) The magnitude of photocurrent measured under bias voltages of 0 V and −6 V varies with the optical power density. (**c**) The fitting curve of the relationship between the photocurrent and optical power density measured under bias voltages of 0 V and −6 V. (**d**) Response time.

$J_{p}$ and $\;J_{d}$ represent the photocurrent density and dark current density, respectively. The relationship between the PDCR and the optical power density of the GNR/Al_2_O_3_/Si photodetector under 0 V and −6 V bias voltages is shown in Figure 3a. At a bias voltage of 0 V and a light power density of 26.1 mW/cm^2^, the highest PDCR of 624.63 was obtained. However, when the bias voltage was −6 V, the highest PDCR decreased to 2.64, attributed to the high dark current at −6 V. This phenomenon indicates that the photodetector is more suitable for detecting weak light in self-powered mode due to the low dark current. Under the 0 V bias voltage, by changing the power intensity of the incident light, the PDCR of the photodetector can be adjusted from 186.096 (0.038 mW/cm^2^) to 624.63 (26.1 mW/cm^2^). It is worth noting that a high PDCR, ~186.096, was achieved at a weak optical power density of 0.038 mW/cm^2^, revealing its ability to detect ultra-weak optical signals. Figure 3b shows the optical power density dependence of the device’s photocurrent under 0 V and −6 V bias voltages, indicating a positive correlation between optical power density and photocurrent.

As shown in Figure 3c, the relationship between optical power density (P) and photocurrent (I_ph_) is described as a power law: $I_{\mathrm{ph}}\propto P^{\theta }$, where θ is an important parameter reflecting the photoelectric performance of the device. The fitted value is much smaller than the ideal factor of 1, indicating the presence of trap states in the device. It is evident that the photocurrent depends on the intensity of light and increases with the increase in light power density, which is consistent with the fact that the number of photo-generated carriers is proportional to the absorbed light flux [45,46,47,48,49].

Response time is another key indicator of photodetectors, with rise time representing the duration of the pulse current increasing from 10% to 90% of the peak value, and decay time defined as the time required to decrease from 90% to 10%, as shown in Figure 3d. GNR/Al_2_O_3_/Si photodetectors exhibit astonishing fast-response characteristics. To evaluate the response speed of the device, an oscilloscope was used to measure the photovoltaic voltage changes within a single laser pulse, as shown in the figure. The rise time (τ_r_) and decay time (τ_d_) are 680 μs and 307.5 μs, respectively, indicating a fast light response speed. The rapid response is due to the strong built-in electric field at the GNR/Al_2_O_3_/Si interface, which promotes the effective separation of photo-generated charge carriers.

In order to further quantitatively evaluate the optoelectronic detection performance of GNR/Al_2_O_3_/Si devices, three key parameters are required, including responsivity (R), detectivity (D*), and external quantum efficiency (EQE):

(4) $$R\;=\;\frac{\left|J_{p}-J_{d}\right|}{P}$$

(5) $$D^{*}=\frac{R}{\sqrt{2eJ_{d}}}$$

(6) $$\mathrm{EQE}=\frac{\mathrm{hcR}}{e\lambda }\;\times \;100\%$$

Among them, P, e, h, c and *λ* are the optical power density, fundamental charge, Planck constant, speed of light in vacuum, and wavelength of laser irradiation, respectively. Generally speaking, the responsivity of photodetectors is a measure of sensitivity, while the detectivity reflects the ability of photodetectors to distinguish weak light signals. The responsivity under 0 V bias varies between 0.03 and 8.71 A/W within the optical power density range of 0.038 to 39.2 mW/cm^2^. The key parameter performance is optimal at an optical power density of 0.038 mW/cm^2^. At this optical power density, the responsivity and external quantum efficiency reach their optimal values of 159.55 A/W and 18,629.37% at −6 V bias, respectively. The detectivity reaches its maximum value of 1.15 × 10^13^ at zero bias. In PN heterojunction-based photodetectors, photo-generated electron–hole pairs can be effectively separated by an internal built-in voltage. Separate charges of opposite polarity will generate opposite Coulomb potentials to balance the built-in potential in the junction. Therefore, the transmission current through the heterojunction will increase, resulting in a higher EQE [50]. In addition, when the device operates under reverse bias, EQE further increases because the larger reverse voltage further reduces the injection barrier, enhances the electric field inside the junction, and causes a sharp increase in the photocurrent [51]. The abnormality of EQE > 100% can be attributed to the trap-assisted carrier multiplication mechanism between different materials in our device structure. When near-infrared light irradiates the device to generate electron-hole pairs, some photo-generated carriers (mainly holes with lower mobility) will be captured by trap states inside the GNR film, at the interface between GNR and Al_2_O_3_, and on the Si surface. They will be localized near the interface for a long time, forming a stable space charge region and an additional built-in electric field, which will prompt the electrode to inject a large number of movable electrons into the active layer. Each photon absorbed can collect multiple carriers to achieve carrier gain. Traps act as charge storage centers in this process to extend carrier lifetime and reduce recombination rate, resulting in a much larger number of collected carriers than incident photons, ultimately leading to an EQE greater than 100%. At the same time, the Al_2_O_3_ insulation layer acts as a barrier and passivation layer, further suppressing dark current and non-radiative recombination, making the gain effect more significant [52,53,54,55,56,57,58,59,60,61].

**Figure 4:**
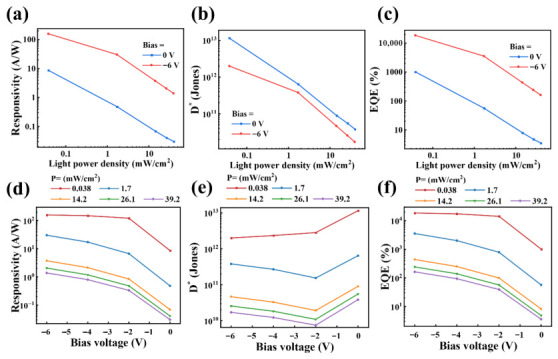
(**a**) Relationship between responsivity and optical power density under 0 V and −6 V bias voltages. (**b**) The responsivity varies with the bias voltage at different optical power densities. (**c**) Relationship between detectivity and optical power density under 0 V and −6 V bias voltages. (**d**) The detectivity varies with bias voltage under different optical power densities. (**e**) Relationship between external quantum efficiency and optical power density under 0 V and −6 V bias voltages. (**f**) The external quantum efficiency varies with bias voltage under different optical power densities.

As shown in Figure 4a,c,e, under bias conditions of 0 V and −6 V, the responsivity, detectivity, and EQE all decrease with increasing optical power. The trap state at the interface is considered to be the cause of this phenomenon. When the trap state saturates at a certain optical power density, further illumination will generate additional free carriers that cannot be captured by the trap, resulting in a shortened average carrier lifetime and decreased performance parameters. Figure 4b,d,f show the curves of the optoelectronic performance under different bias voltages at different optical power densities. From Figure 4b,f, we can understand that at a fixed optical power density, the responsivity and EQE both increase with the increase in bias voltage. This is because the dark current increases sharply under high bias voltage, while the maximum increment of photocurrent is limited by the upper limit of the total amount of photo-generated carriers under constant optical power. The interfacial electric field of the heterojunction intensifies with increasing reverse bias voltage. This enhancement accelerates the separation of photo-generated carriers, mitigates carrier recombination losses, and thus improves carrier transport efficiency. Despite the fact that interfacial defects on the device surface may induce an increase in leakage current, the growth rate of photocurrent remains significantly higher than that of dark current under constant optical power density. Owing to the dominant contribution of photocurrent, both the responsivity and EQE of the device increase monotonically with the elevation of reverse bias voltage.

Figure 4d shows a different situation. Under weak light conditions with an optical power density of 0.038 mW/cm^2^, the detectivity decreases monotonically with increasing bias voltage. In contrast, under stronger optical power density conditions, the detectivity peaks at 0 V. With a further increase in bias voltage, the detectivity initially declines and then exhibits a gradual upward trend. This phenomenon can be attributed to the following mechanism: under weak light illumination, the dark current increases sharply as the bias voltage rises from 0 V to −2 V, followed by a slow upward trend when the bias voltage is further increased from −2 V to −6 V. Given the limited photon number under weak light conditions, increasing the bias voltage only marginally enhances the efficiency of photo-generated carrier separation. As a result, the increment in responsivity is far less pronounced than the elevation in dark current, which ultimately leads to a continuous reduction in detectivity. After increasing the optical power, the operation of the device at 0 V still depends on the built-in electric field of the heterojunction. The core reason for the highest detectivity at this time is that the dark current is extremely small, and the responsivity remains at an effective level. The combination of the two makes the detectivity reach its peak. As the reverse bias increases from 0 V to −2 V, the detectivity drops sharply. This is due to the surge in dark current at low bias. However, the enhancement of the electric field has limited effect on carrier separation, and the responsivity growth is relatively slow. Finally, it leads to a decrease in detectivity due to the dominance of dark current. As the bias voltage further increases, the interface electric field strength significantly increases, completely separating the uncaptured photo-generated carriers under low bias voltage and accelerating the release of carriers from trap states. At the same time, the growth rate of dark current slows down significantly, and the increase in responsivity far exceeds the influence of dark current, gradually increasing the detectivity. For higher optical power densities, due to the approaching saturation of photo-generated carriers, the increase in responsivity slows down relatively, but still exceeds the influence of dark current. Therefore, the detectivity still shows a trend of first decreasing and then recovering, but the recovery amplitude is gentler than that of medium optical power, ultimately presenting the result shown in Figure 4d.

**Figure 5:**
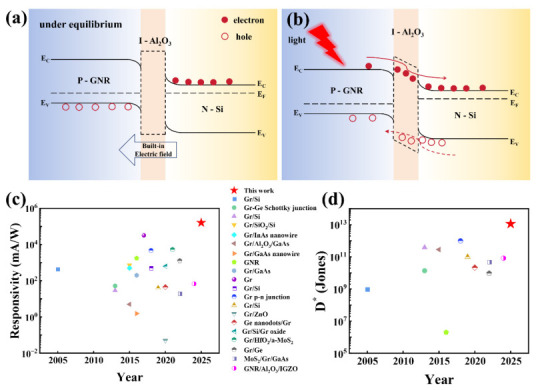
(**a**) Energy band structure of the GNR/Al_2_O_3_/Si heterojunction under equilibrium state. (**b**) Energy band structure and carrier transport behavior of the GNR/Al_2_O_3_/Si heterojunction under illumination. (**c**) Comparative analysis of responsivity between this work and reported graphene-based heterojunction photodetectors in the literature. (**d**) Comparative analysis of detectivity between this work and reported graphene-based heterojunction photodetectors in the literature.

In order to clarify the physical mechanism of high responsivity and enhanced photoresponsivity in GNR/Al_2_O_3_/Si heterojunctions with an Al_2_O_3_ interface layer, the energy band structure and carrier transport behavior of the device are illustrated in Figure 5a,b. As depicted in Figure 5a, in the p-GNR/Al_2_O_3_/n-Si heterojunction, electrons diffuse from the Si layer to the GNR layer, while holes do the opposite. When reaching thermal equilibrium, a depletion region is formed at the interface, and the energy band bends upward near the heterojunction, forming an intrinsic electric field pointing from Si to GNR. Figure 5b highlights the generation, separation, and recombination of electron–hole pairs under 1064 nm laser irradiation. When light is irradiated onto the PN junction, the energy of photons is absorbed by the semiconductor material, generating electron–hole pairs. In theory, photo-generated carriers are separated by an internal electric field, and photo-generated electrons flow towards the Si side, while photo-generated holes flow towards the GNR side, thereby generating photocurrent. However, in practical devices, the non-dense surface of GNR thin films and residual highly conductive CNT can generate many reverse current leakage paths, resulting in high dark current. In GNR/Al_2_O_3_/Si devices, Al_2_O_3_ acts as a Schottky barrier, which can significantly reduce interface carrier recombination, effectively block leakage paths, and promote effective hole tunneling, thereby enhancing photocurrent and suppressing dark current [62,63,64] because holes must tunnel through a potential barrier under reverse bias to reach GNR from Si. This barrier causes hole accumulation at the Si interface, generating corresponding induced electrons at the GNR interface, forming an induced electric field in the same direction as the built-in electric field, enhancing the effect of the built-in electric field [24]. By reducing the reverse dark current of Al_2_O_3_, the hole density in the GNR valence band is lowered. In addition, GNR with an open bandgap plays a crucial role in photon absorption, participating in the absorption of photons and improving the photoelectric detection performance [65]. Figure 5c,d shows the various performance indicators of graphene-related heterojunction photodetectors previously reported in the literature. Comparison shows that photodetectors using GNR/Al_2_O_3_/Si heterojunctions outperform most existing graphene-related devices in terms of individual or combined performance indicators [5,6,22,37,66,67,68,69,70,71,72,73,74,75,76,77,78,79,80,81,82]. Table S1 details the comparison of various optoelectronic performance parameters between this work and previously reported graphene-related photodetectors.

**Figure 6:**
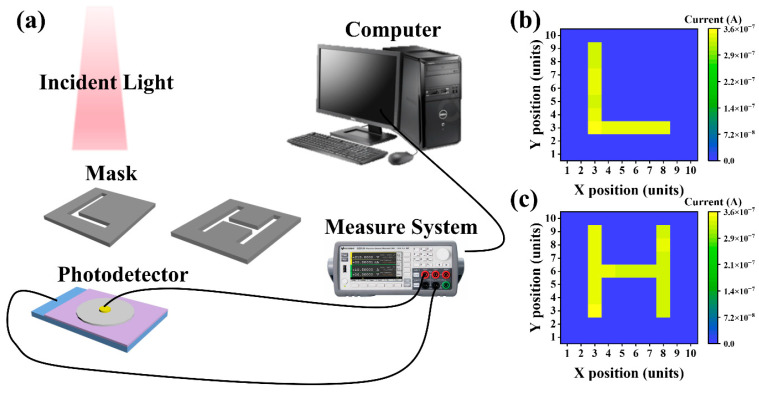
(**a**) Schematic diagram of imaging testing system. (**b**,**c**) Imaging results of GNR/Al_2_O_3_/Si photodetector.

To verify the application potential of the GNR/Al_2_O_3_/Si vertical heterojunction photodetector in practical scenarios, this work constructed a simple single-pixel scanning imaging system to preliminarily evaluate the near-infrared imaging capability of the device. The optical path structure of the imaging system is shown in Figure 6a. The 1064 nm near-infrared laser serves as the illumination source, and a black mask with engraved hollow letters “L” and “H” patterns is placed in the optical path. By moving the mask plate, the photodetector is tested point-by-point in the two-dimensional plane. The photocurrent signal output by the detector is collected by a semiconductor parameter analyzer and transmitted to the computer. Through data processing, a simple image is finally formed.

The imaging experiment was conducted in the zero-bias-voltage self-powered mode. Figure 6b shows the near-infrared imaging results. When the hollow areas on the mask are exposed to infrared light, the detector generates a significant photocurrent response, corresponding to the bright area in the image; while the areas blocked by the black mask only generate extremely low dark currents, corresponding to the dark background in the image. The contour edges of the letters “L” and “H” are clearly distinguishable, proving that the device has good spatial resolution in the near-infrared wavelength range. This is of great reference value for the development of portable and low-power near-infrared imaging systems.

To further evaluate the long-term stability and practical application potential of the device, we conducted dynamic current response retesting on the GNR/Al_2_O_3_/Si photodetector after 6 months of storage, as shown in Figure S6. The results show that after long-term storage at room temperature, the response characteristics of the device remain good under 1064 nm near-infrared light with similar optical power density as before, and there is no significant attenuation in the value of photocurrent, responsivity, and repeatability. This result confirms that the GNR/Al_2_O_3_/Si vertical heterojunction structure has excellent environmental stability. The interface defects and carrier transport channels do not show significant degradation after long-term storage, which can provide strong guarantees for the reliable operation of this type of near-infrared photodetector in long-term service scenarios and further support its practical application value in low-power, high-stability near-infrared detection systems.

## 4. Conclusions

In summary, we have prepared a photodetector made of p-GNR/Al_2_O_3_/n-Si heterojunction obtained by dissociating DWCNTs with a Al_2_O_3_ interface layer, which exhibits good optoelectronic performance under laser irradiation at a wavelength of 1064 nm. In the device, photon absorption and carrier separation mainly occur in the PN junction region, achieving efficient photoelectric conversion and fast response time. The photodetector exhibits excellent responsivity performance. At a bias voltage of −6 V, the device exhibits a responsivity of 159.55 A/W, a detectivity of 2.01 × 10^12^ Jones, and an EQE of 18,629.37%. Even under 0 V bias, it has high responsivity and detectivity of 8.71 A/W and 1.15 × 10^13^ Jones, as well as a response time of 307.5 μs, indicating its potential for self-powering. These results indicate that GNR-based photodetectors derived from dissociated DWCNTs are promising candidates for future high-performance and low-cost photodetectors.

## Data Availability

The data supporting this article have been included as part of the Supplementary Information.

## Supplementary Materials

The following supporting information can be downloaded at https://www.mdpi.com/article/10.3390/nano16100625/s1. Figure S1. Optical micrograph of GNR/Al_2_O_3_/Si photodetector. Figure S2. (**a**) Atomic force microscope image of the GNR thin film; (**b**) Three-dimensional morphology of the GNR film. Figure S3. (**a**) Relationship between photocurrent and bias voltage of GNR/Si photodetector at different optical power densities. (**b**) The fitting curve of the relationship between the photocurrent and optical power density measured under bias voltages of 0 V and −6 V. Dynamic current response of the photodetectors at different optical power densities with bias voltages of (**c**) 0 V and (**d**) −6 V. Dynamic current response of photodetectors at different bias voltages with optical power densities of (**e**) 0.003 mW/cm^2^ and (**f**) 10.21 mW/cm^2^. Figure S4. (**a**) Relationship between photocurrent and bias voltage of GNR/Al_2_O_3_/Si photodetector with 5 nm Al_2_O_3_ at different optical power densities. (**b**) The fitting curve of the relationship between the photocurrent and optical power density measured under bias voltages of 0 V and −6 V. Dynamic current response of the photodetectors at different optical power densities with bias voltages of (**c**) 0 V and (**d**) −6 V. Dynamic current response of photodetectors at different bias voltages with optical power densities of (**e**) 0.008 mW/cm^2^ and (**f**) 1.65 mW/cm^2^. Figure S5. (**a**) Relationship between photocurrent and bias voltage of GNR/Al_2_O_3_/Si photodetector with 15 nm Al_2_O_3_ at different optical power densities. (**b**) The fitting curve of the relationship between the photocurrent and optical power density measured under bias voltages of 0 V and −6 V. Dynamic current response of the photodetectors at different optical power densities with bias voltages of (**c**) 0 V and (**d**) −6 V. Dynamic current response of photodetectors at different bias voltages with optical power densities of (**e**) 0.028 mW/cm^2^ and (**f**) 55 mW/cm^2^. Figure S6. the dynamic response curve of the GNR/Al_2_O_3_/Si photodetector after being placed for 6 months. Table S1. Comparison of the performance of the photodetector in this paper with other graphene-related photodetectors.

## References

[1] Wang J., Gudiksen M.S., Duan X., Cui Y., Lieber C.M. (2001). Highly Polarized Photoluminescence and Photodetection from Single Indium Phosphide Nanowires. Science.

[2] Kind H., Yan H., Messer B., Law M., Yang P. (2002). Nanowire Ultraviolet Photodetectors and Optical Switches. Adv. Mater..

[3] Peng L., Hu L., Fang X. (2014). Energy Harvesting for Nanostructured Self-Powered Photodetectors. Adv. Funct. Mater..

[4] Hsu C.L., Chang S.J. (2014). Doped ZnO 1D Nanostructures: Synthesis, Properties, and Photodetector Application. Small.

[5] Miao J., Hu W., Guo N., Lu Z., Liu X., Liao L., Chen P., Jiang T., Wu S., Ho J.C. (2015). High-Responsivity Graphene/InAs Nanowire Heterojunction Near-Infrared Photodetectors with Distinct Photocurrent On/Off Ratios. Small.

[6] Zeng L.-H., Wang M.-Z., Hu H., Nie B., Yu Y.-Q., Wu C.-Y., Wang L., Hu J.-G., Xie C., Liang F.-X. (2013). Monolayer Graphene/Germanium Schottky Junction As High-Performance Self-Driven Infrared Light Photodetector. ACS Appl. Mater. Interfaces.

[7] Guo Q., Pospischil A., Bhuiyan M., Jiang H., Tian H., Farmer D., Deng B., Li C., Han S.-J., Wang H. (2016). Black Phosphorus Mid-Infrared Photodetectors with High Gain. Nano Lett..

[8] Gong X., Tong M., Xia Y., Cai W., Moon J.S., Cao Y., Yu G., Shieh C.L., Nilsson B., Heeger A.J. (2009). High-Detectivity Polymer Photodetectors with Spectral Response from 300 nm to 1450 nm. Science.

[9] Zhou X., Gan L., Zhang Q., Xiong X., Li H., Zhong Z., Han J., Zhai T. (2016). High performance near-infrared photodetectors based on ultrathin SnS nanobelts grown via physical vapor deposition. J. Mater. Chem. C.

[10] Xie C., Liu C.K., Loi H.L., Yan F. (2019). Perovskite-Based Phototransistors and Hybrid Photodetectors. Adv. Funct. Mater..

[11] Long M., Wang P., Fang H., Hu W. (2018). Progress, Challenges, and Opportunities for 2D Material Based Photodetectors. Adv. Funct. Mater..

[12] Bullock J., Amani M., Cho J., Chen Y.-Z., Ahn G.H., Adinolfi V., Shrestha V.R., Gao Y., Crozier K.B., Chueh Y.-L. (2018). Polarization-resolved black phosphorus/molybdenum disulfide mid-wave infrared photodiodes with high detectivity at room temperature. Nat. Photonics.

[13] Miao J., Zhang F. (2019). Recent progress on highly sensitive perovskite photodetectors. J. Mater. Chem. C.

[14] Xia F., Wang H., Xiao D., Dubey M., Ramasubramaniam A. (2014). Two-dimensional material nanophotonics. Nat. Photonics.

[15] Tian W., Zhang C., Zhai T., Li S.L., Wang X., Liu J., Jie X., Liu D., Liao M., Koide Y. (2014). Flexible Ultraviolet Photodetectors with Broad Photoresponse Based on Branched ZnS-ZnO Heterostructure Nanofilms. Adv. Mater..

[16] Soci C., Zhang A., Xiang B., Dayeh S.A., Aplin D.P.R., Park J., Bao X.Y., Lo Y.H., Wang D. (2007). ZnO nanowire UV photodetectors with high internal gain. Nano Lett..

[17] Xu H., Wu J., Feng Q., Mao N., Wang C., Zhang J. (2014). High Responsivity and Gate Tunable Graphene-MoS2 Hybrid Phototransistor. Small.

[18] Li X., Gao C., Duan H., Lu B., Wang Y., Chen L., Zhang Z., Pan X., Xie E. (2012). High-Performance Photoelectrochemical-Type Self-Powered UV Photodetector Using Epitaxial TiO2/SnO2 Branched Heterojunction Nanostructure. Small.

[19] Yang L., Wang S., Zeng Q., Zhang Z., Peng L.M. (2013). Carbon Nanotube Photoelectronic and Photovoltaic Devices and their Applications in Infrared Detection. Small.

[20] Bie Y.Q., Liao Z.M., Zhang H.Z., Li G.R., Ye Y., Zhou Y.B., Xu J., Qin Z.X., Dai L., Yu D.P. (2010). Self-Powered, Ultrafast, Visible-Blind UV Detection and Optical Logical Operation based on ZnO/GaN Nanoscale p-n Junctions. Adv. Mater..

[21] Rogalski A. (2002). Infrared detectors: An overview. Infrared Phys. Technol..

[22] Lv P., Zhang X., Zhang X., Deng W., Jie J. (2013). High-Sensitivity and Fast-Response Graphene/Crystalline Silicon Schottky Junction-Based Near-IR Photodetectors. IEEE Electron Device Lett..

[23] Yu K., Chen J. (2008). Enhancing Solar Cell Efficiencies through 1-D Nanostructures. Nanoscale Res. Lett..

[24] Sun Y., Wang M., Zheng X., Li Z., Han N., Li M., Wang Z., Han L., Ning Y., Fatima S. (2025). Superior Self-Powered Infrared Photodetector via Semiconducting Graphene-Nanoribbons-Based Vertical Heterojunctions. Appl. Phys. Rev..

[25] Buscema M., Island J.O., Groenendijk D.J., Blanter S.I., Steele G.A., van der Zant H.S., Castellanos-Gomez A. (2015). Photocurrent generation with two-dimensional van der Waals semiconductors. Chem. Soc. Rev..

[26] Xie C., Mak C., Tao X., Yan F. (2016). Photodetectors Based on Two-Dimensional Layered Materials Beyond Graphene. Adv. Funct. Mater..

[27] Xie C., Wang Y., Zhang Z.-X., Wang D., Luo L.-B. (2018). Graphene/Semiconductor Hybrid Heterostructures for Optoelectronic Device Applications. Nano Today.

[28] Xie C., Zeng L., Zhang Z., Tsang Y.-H., Luo L., Lee J.-H. (2018). High-performance broadband heterojunction photodetectors based on multilayered PtSe2 directly grown on a Si substrate. Nanoscale.

[29] Novoselov K.S., Geim A.K., Morozov S.V., Jiang D., Zhang Y., Dubonos S.V., Grigorieva I.V., Firsov A.A. (2004). Electric Field Effect in Atomically Thin Carbon Films. Science.

[30] Kim W.Y., Kim K.S. (2008). Prediction of very large values of magnetoresistance in a graphene nanoribbon device. Nat. Nanotechnol..

[31] Senkovskiy B.V., Pfeiffer M., Alavi S.K., Bliesener A., Zhu J., Michel S., Fedorov A.V., German R., Hertel D., Haberer D. (2017). Making Graphene Nanoribbons Photoluminescent. Nano Lett..

[32] Ma F., Guo Z., Xu K., Chu P.K. (2012). First-principle study of energy band structure of armchair graphene nanoribbons. Solid State Commun..

[33] Yang L., Park C.-H., Son Y.-W., Cohen M.L., Louie S.G. (2007). Quasiparticle Energies and Band Gaps in Graphene Nanoribbons. Phys. Rev. Lett..

[34] Han Y., Jiao S., Jing J., Chen L., Shi Z., Rong P., Wang D., Gao S., He W., Wang J. (2023). Vertical heterojunction photodetector with self-powered broadband response and high performance. Chem. Eng. J..

[35] Liu T.W., Zhao Z., Cao R., Liu Y.Y., Jiang X. (2025). Reliability challenges of gate dielectric materials in transistors. Inf. Funct. Mater..

[36] Liu W., Yu Y., Peng M., Zheng Z., Jian P., Wang Y., Zou Y., Zhao Y., Wang F., Wu F. (2023). Integrating 2D layered materials with 3D bulk materials as van der Waals heterostructures for photodetections: Current status and perspectives. InfoMat.

[37] Ye X., Zheng X., Han L., Sun Y., Wang L., Li Z., Liu W., Liu B., Han N., Khan S. (2024). High Performance Self-Powered Photodetectors Based on Graphene Nanoribbons/Al_2_O_3_/InGaZnO Heterojunctions. IEEE Photonics J..

[38] Gamiño-Barocio I., Vázquez-Vázquez E.F., Hernández-Rodríguez Y.M., Cigarroa-Mayorga O.E. (2024). Tuning the Charge Transfer in MWCNTs via the Incorporation of ZnONPs and AgNPs: The Role of Carbon Binding with ZnO/Ag Heterostructures in Reactive Species Formation. Nanomaterials.

[39] Guo Y. (2020). Synthetic and Crystallographic Study of Various Functional Low-Dimensional Perovskites. Ph.D. Thesis.

[40] Zhang X., Tang Z., Wang Y., Zhao F., He Q., Ren A., Zhang J., Ren W., Wu J. (2025). High-Fundamental-Mode Output of 1064 nm Vertical-Cavity Surface-Emitting Laser Using Double Embedded Antiresonant Oxide Islands. Inf. Funct. Mater..

[41] Cigarroa-Mayorga O.E., Gallardo-Hernández S., Talamás-Rohana P. (2021). Tunable Raman scattering enhancement due to self-assembled Au nanoparticles layer on porous AAO: The influence of the alumina support. Appl. Surf. Sci..

[42] Xiang G., Zhang J., Yue Z., Zhang X., Song C., Ding B., Wang L., Wang Y., He H., Wang H. (2023). High Storage and Operational Stability Self-Powered UV Photodetector Based on p-CuI/n-GaN Heterojunction Prepared by Thermal Evaporation Method. Appl. Surf. Sci..

[43] An X., Liu F., Jung Y.J., Kar S. (2013). Tunable Graphene–Silicon Heterojunctions for Ultrasensitive Photodetection. Nano Lett..

[44] Xu C.H., Luo S.H., Wang Y., Shi X.F., Fu C., Wang J., Wu C.Y., Luo L.B. (2023). Bias-Selectable Si Nanowires/PbS Nanocrystalline Film n–n Heterojunction for NIR/SWIR Dual-Band Photodetection. Adv. Funct. Mater..

[45] Qiao S., Liu Y., Liu J., Fu G., Wang S. (2021). High-Responsivity, Fast, and Self-Powered Narrowband Perovskite Heterojunction Photodetectors with a Tunable Response Range in the Visible and Near-Infrared Region. ACS Appl. Mater. Interfaces.

[46] Wu D., Guo J., Wang C., Ren X., Chen Y., Lin P., Zeng L., Shi Z., Li X.J., Shan C.-X. (2021). Ultrabroadband and High-Detectivity Photodetector Based on WS2/Ge Heterojunction through Defect Engineering and Interface Passivation. ACS Nano.

[47] Wang Z., Geng X., Jin J., Zhang P., Zhang L., Li Y., Du J., He Y., Zhang M., Peng J. (2024). Self-Powered Photodetector Based on Perovskite/Ge Heterojunction with High Responsivity. Adv. Mater. Technol..

[48] Li X., Wu S.E., Wu D., Zhao T., Lin P., Shi Z., Tian Y., Li X., Zeng L., Yu X. (2023). In situ construction of PtSe2/Ge Schottky junction array with interface passivation for broadband infrared photodetection and imaging. InfoMat.

[49] Xiao Y., Deng K., Zhang K., Xu X., Li Q., Zhang T., Guo J., Lu H., Wang P., Hu W. (2024). On-Chip Room-Temperature Operated Short-Wavelength-Infrared Si:S Photodetector with a Vertical Junction. Adv. Funct. Mater..

[50] Zhao Y., Cho J., Choi M., Ó Coileáin C., Arora S., Hung K.-M., Chang C.-R., Abid M., Wu H.-C. (2022). Light-Tunable Polarity and Erasable Physisorption-Induced Memory Effect in Vertically Stacked InSe/SnS2 Self-Powered Photodetector. ACS Nano.

[51] Kublitski J., Fischer A., Xing S., Baisinger L., Bittrich E., Spoltore D., Benduhn J., Vandewal K., Leo K. (2021). Enhancing sub-bandgap external quantum efficiency by photomultiplication for narrowband organic near-infrared photodetectors. Nat. Commun..

[52] Yang B., Gao W., Li H., Gao P., Yang M., Pan Y., Wang C., Yang Y., Huo N., Zheng Z. (2023). Visible and Infrared Photodiode Based on γ-InSe/Ge Van der Waals Heterojunction for Polarized Detection and Imaging. Nanoscale.

[53] Wang Y., Zhu L., Wang T., Hu Y., Deng Z., Cui Q., Lou Z., Hou Y., Teng F. (2018). Fast and Sensitive Polymer Photodetectors with Extra High External Quantum Efficiency and Large Linear Dynamic Range at Low Working Voltage Bias. Org. Electron..

[54] Zhao Z., Xu C., Ma Y., Yang K., Liu M., Zhu X., Zhou Z., Shen L., Yuan G., Zhang F. (2022). Ultraviolet Narrowband Photomultiplication Type Organic Photodetectors with Fabry−Pérot Resonator Architecture. Adv. Funct. Mater..

[55] Bansal S., Sharma K., Jain P., Sardana N., Kumar S., Gupta N., Singh A.K. (2018). Bilayer Graphene/HgCdTe Based Very Long Infrared Photodetector with Superior External Quantum Efficiency, Responsivity, and Detectivity. RSC Adv..

[56] Zhao X., Huang L., Wang Y., Zhu X., Li L., Li G., Sun W. (2020). Interface Engineering for Gain Perovskite Photodetectors with Extremely High External Quantum Efficiency. RSC Adv..

[57] Xiao Z., Xu H., Liang W., Wu B., Shi Y., Deng H., Lan Y., Long Y. (2021). Effective Film Surface Treatment for Improving External Quantum Efficiency of Photomultiplication Type Organic Photodetector. High Perform. Polym..

[58] Liu M.-y., Wang J., Yang K.-x., Liu M., Zhao Z.-j., Zhang F.-j. (2021). Broadband Photomultiplication Organic Photodetectors. Phys. Chem. Chem. Phys..

[59] Zhao X., Jiang D., Zhao M., Duan Y. (2021). Avalanche Effect and High External Quantum Efficiency in MgZnO/Au/ZnO Sandwich Structure Photodetector. Adv. Opt. Mater..

[60] Csucker J., Didier E., Pedro Ferreira Assunção J., Rentsch D., Kothandaraman R., Bachmann D., Shorubalko I., Nüesch F., Hany R., Bauer M. (2025). Squaraine Dyes for Organic Photomultiplication Photodetectors with 220% External Quantum Efficiency at 1240 nm. Adv. Sci..

[61] Min L., Sun H., Guo L., Zhou Y., Wang M., Cao F., Li L. (2024). Pyroelectric-Accelerated Perovskite Photodetector for Picosecond Light Detection and Ranging. Adv. Mater..

[62] Kim C., Yoo T.J., Chang K.E., Kwon M.G., Hwang H.J., Lee B.H. (2021). Highly Responsive Near-Infrared Photodetector with Low Dark Current Using Graphene/Germanium Schottky Junction with Al_2_O_3_ Interfacial Layer. Nanophotonics.

[63] Das S., Pandey D., Thomas J., Roy T. (2018). The Role of Graphene and Other 2D Materials in Solar Photovoltaics. Adv. Mater..

[64] Rehman M.A., Akhtar I., Choi W., Akbar K., Farooq A., Hussain S., Shehzad M.A., Chun S.-H., Jung J., Seo Y. (2018). Influence of an Al_2_O_3_ Interlayer in a Directly Grown Graphene-Silicon Schottky Junction Solar Cell. Carbon.

[65] Wang M., Zheng X., Ye X., Liu W., Zhang B., Zhang Z., Zhai R., Ning Y., Li H., Song A. (2023). High-Performance Photodetectors Based on Semiconducting Graphene Nanoribbons. Nano Lett..

[66] Wang C., Dong Y., Lu Z., Chen S., Xu K., Ma Y., Xu G., Zhao X., Yu Y. (2019). High Responsivity and High-Speed 1.55 μm Infrared Photodetector from Self-Powered Graphene/Si Heterojunction. Sens. Actuators A Phys..

[67] Periyanagounder D., Gnanasekar P., Varadhan P., He J.-H., Kulandaivel J. (2018). High Performance, Self-Powered Photodetectors Based on a Graphene/Silicon Schottky Junction Diode. J. Mater. Chem. C.

[68] Novoselov K.S., Geim A.K., Morozov S.V., Jiang D., Katsnelson M.I., Grigorieva I.V., Dubonos S.V., Firsov A.A. (2005). Two-Dimensional Gas of Massless Dirac Fermions in Graphene. Nature.

[69] Wang G., Zhang M., Chen D., Guo Q., Feng X., Niu T., Liu X., Li A., Lai J., Sun D. (2018). Seamless Lateral Graphene P–N Junctions Formed by Selective in situ Doping for High-Performance Photodetectors. Nat. Commun..

[70] Li X., Zhu M., Du M., Lv Z., Zhang L., Li Y., Yang Y., Yang T., Li X., Wang K. (2015). High Detectivity Graphene-Silicon Heterojunction Photodetector. Small.

[71] Wu Y., Yan X., Zhang X., Ren X. (2016). A Monolayer Graphene/GaAs Nanowire Array Schottky Junction Self-Powered Photodetector. Appl. Phys. Lett..

[72] Chen D., Xin Y., Lu B., Pan X., Huang J., He H., Ye Z. (2020). Self-Powered Ultraviolet Photovoltaic Photodetector Based on Graphene/ZnO Heterostructure. Appl. Surf. Sci..

[73] Kwon M.G., Kim C., Chang K.E., Yoo T.J., Kim S.-Y., Hwang H.J., Lee S., Lee B.H. (2022). Performance Enhancement of Graphene/Ge Near-Infrared Photodetector by Modulating the Doping Level of Graphene. APL. Photonics.

[74] Qu J., Chen J. (2022). Graphene/GaAs Schottky Junction Near-Infrared Photodetector With a MoS2 Quantum Dots Absorption Layer. IEEE Trans. Electron Devices.

[75] Huang Z., Liu J., Zhang T., Jin Y., Wang J., Fan S., Li Q. (2021). Interfacial Gated Graphene Photodetector with Broadband Response. ACS Appl. Mater. Interfaces.

[76] Wang Y., Yang S., Lambada D.R., Shafique S. (2020). A Graphene-Silicon Schottky Photodetector with Graphene Oxide Interlayer. Sens. Actuators A Phys..

[77] Yu J., Zhong J., Kuang X., Zeng C., Cao L., Liu Y., Liu Z. (2020). Dynamic Control of High-Range Photoresponsivity in a Graphene Nanoribbon Photodetector. Nanoscale Res. Lett..

[78] Liu Y., Xia Q., He J., Liu Z. (2017). Direct Observation of High Photoresponsivity in Pure Graphene Photodetectors. Nanoscale Res. Lett..

[79] Yu X., Dong Z., Liu Y., Liu T., Tao J., Zeng Y., Yang J.K.W., Wang Q.J. (2016). A High Performance, Visible to Mid-Infrared Photodetector Based on Graphene Nanoribbons Passivated with HfO2. Nanoscale.

[80] Li X., Lin S., Lin X., Xu Z., Wang P., Zhang S., Zhong H., Xu W., Wu Z., Fang W. (2016). Graphene/h-BN/GaAs Sandwich Diode as Solar Cell and Photodetector. Opt. Express.

[81] Luo L.-B., Hu H., Wang X.-H., Lu R., Zou Y.-F., Yu Y.-Q., Liang F.-X. (2015). A Graphene/GaAs Near-Infrared Photodetector Enabled by Interfacial Passivation with Fast Response and High Sensitivity. J. Mater. Chem. C.

[82] Gao M., Tian Z., Tang S., Han X., Zhang M., Xue Z., Zhu W., Mei Y., Chu P.K., Wang G. (2020). Ambipolar Plasmon-Enhanced Photodetector Built on Germanium Nanodots Array/Graphene Hybrid. Adv. Mater. Interfaces.

