# Supplementary material for: High-Performance Infrared Photodetectors Based on Graphene Nanoribbon Vertical Heterojunctions via Dissociated Double-Walled Carbon Nanotubes
Source: Nanomaterials (Basel). 2026 May 19;16(10):625. doi: 10.3390/nano16100625 (PMC13210341; doi:10.3390/nano16100625)
Supplement: Supplementary file 1 [file nanomaterials-16-00625-s001.zip › nanomaterials-4295767-supplementary.pdf]

# Supplementary Information for

# High-performance Infrared Photodetectors Based on Graphene Nanoribbon Vertical Heterojunctions via Dissociated Dou-ble-Walled Carbon Nanotubes

Ziheng Li[1], Yu Sun[1*], Muyang Li[1], Nan Han[1], Zeyuan Wang[1], Jihui Fan[1], Hui Zhou[1], Xiaoqing Jiang[2], Jie Li[2], Yafei Ning[1,3], Klaus Leifer[4], Mingyang Wang[5], Ming Gao[1,6*], Hu Li[1,3*] and Aimin Song[7,8]

1 Shandong Key Laboratory of Next-Generation Semiconductor Technology and Systems, School of Integrated Circuits, Shandong University, Jinan 250101, China.

2 Shandong Hi-Speed Information Group Co., Ltd., Jinan 250102, China.

3 Shenzhen Research Institute, Shandong University, Shenzhen 518063, China.

4 Department of Engineering Sciences, Uppsala University, 75121 Uppsala, Sweden.

5 State Key Laboratory of Electronic Thin Films and Integrated Devices, Institute of Fundamental and Frontier Sciences, University of Electronic Science and Technology of China, Chengdu 611731, China.

6 Academy of Intelligent Innovation, Shandong University, Jinan 250101, China.

7 Institute of Nanoscience and Applications, Southern University of Science and Technology, Shenzhen 518055, China.

8 Department of Electrical and Electronic Engineering, University of Manchester, Manchester M139PL, UK.

Corresponding to: 202320389@mail.sdu.edu.cn (Y.S.); gaomingsdu@sdu.edu.cn (M.G.); hu.li@sdu.edu.cn (H.L.);

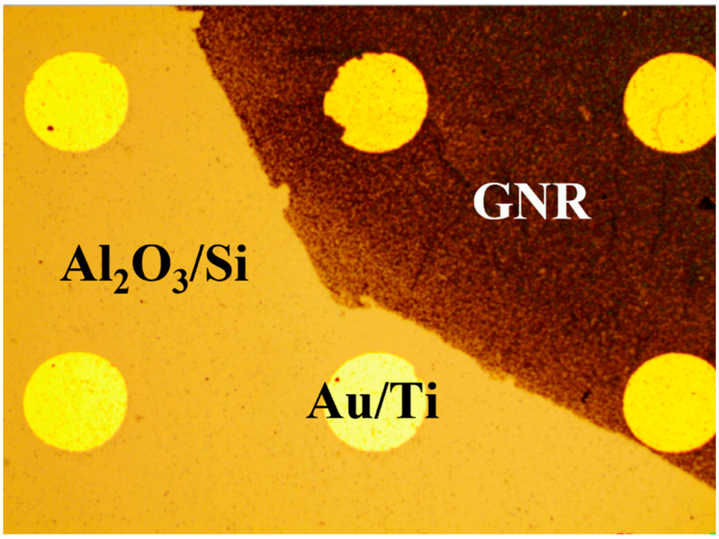


**Figure S1. Optical micrograph of GNR/$Al_2O_3$/Si photodetector.**

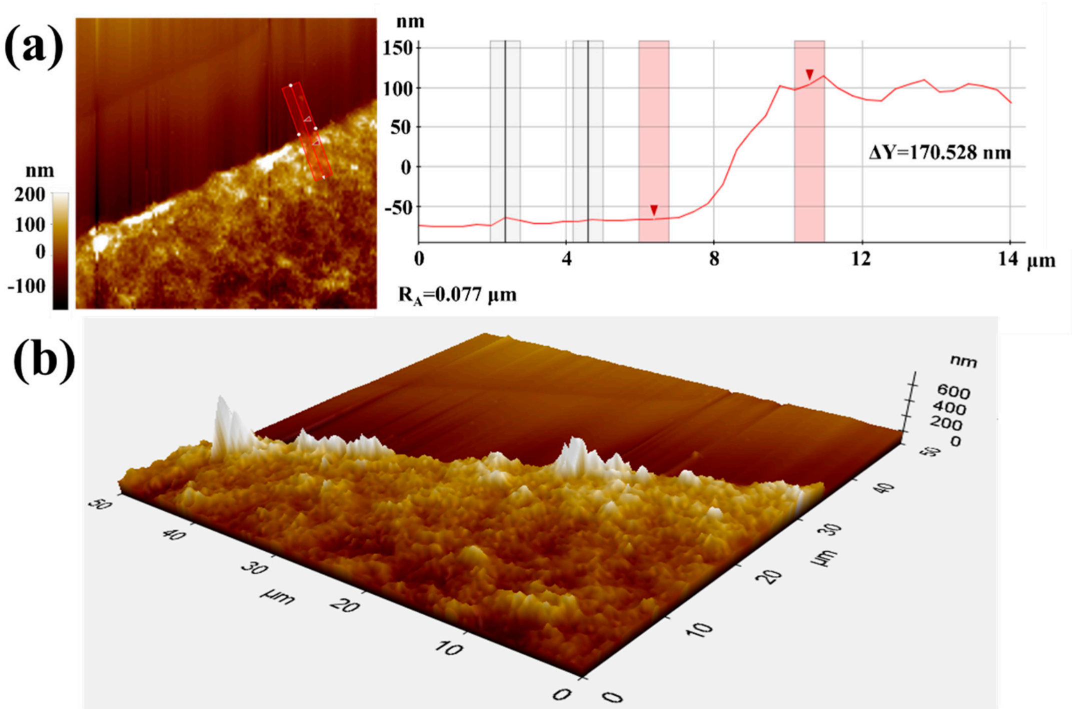


**Figure S2. (a) Atomic force microscope image of the GNR thin film;(b) Three-dimensional morphology of the GNR film.**

Figure S2. (a) shows the surface morphology and thickness characterization of GNR thin films transferred onto substrates and annealed using atomic force microscopy (AFM). Figure S2. (a) shows that the GNR film exhibits uneven surface undulations, which are due to the network structure characteristics formed by the interweaving and stacking of nanoribbons. The clear boundary of the film edge and the obvious step contrast with the substrate indicate that the GNR film maintained good structural integrity during the wet transfer process, without obvious tearing or agglomeration phenomena. The surface roughness of the area was measured to be approximately 0.077 μm. The curve on the right side of Figure S2. (a) shows the average height profile curve within the red marked box on the left. The black box area is selected as the reference

plane of the substrate to eliminate systematic errors caused by sample tilt. The two red arrows indicate the positions corresponding to the substrate and film edge areas, respectively. From the curve data, it can be read that the average thickness of the GNR film edge is about 170 nm. By comparing with the 150 nm thickness measured in the TEM characterization of Figure 1(b), it is indicated that the thickness at the edge of the film is slightly greater than that at the center, which conforms to the typical thickness distribution pattern.

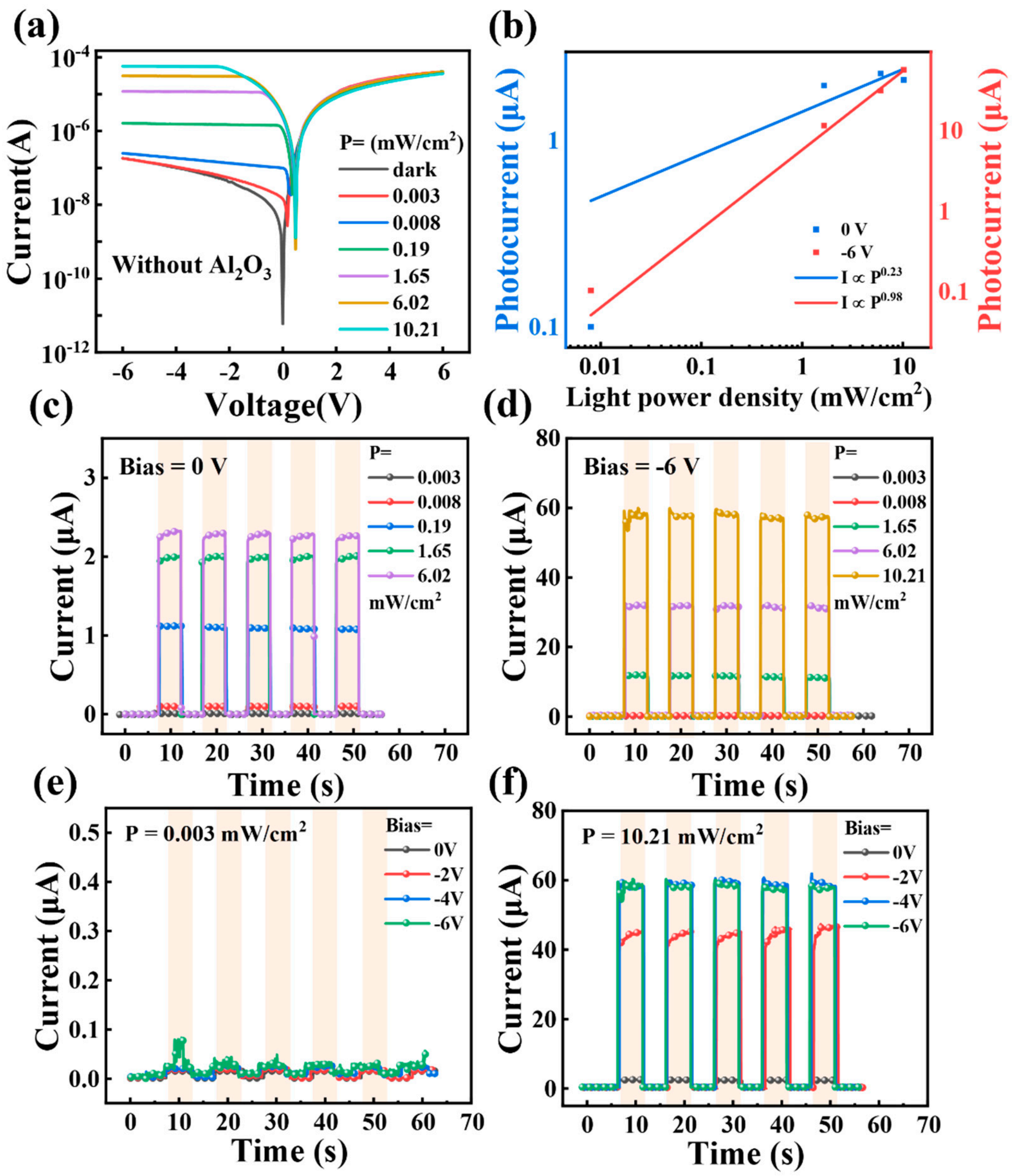


**Figure S3. (a) Relationship between photocurrent and bias voltage of GNR/Si photodetector at different optical power densities. (b) The fitting curve of the relationship between the photocurrent and optical power density measured under bias voltages of 0 V and −6 V. Dynamic current response of the photodetectors at different optical power densities with bias voltages of (c) 0 V and (d) -6 V. Dynamic current response of photodetectors at different bias voltages with optical power densities of (e) 0.003 mW/cm$^2$ and (f) 10.21 mW/cm$^2$.**

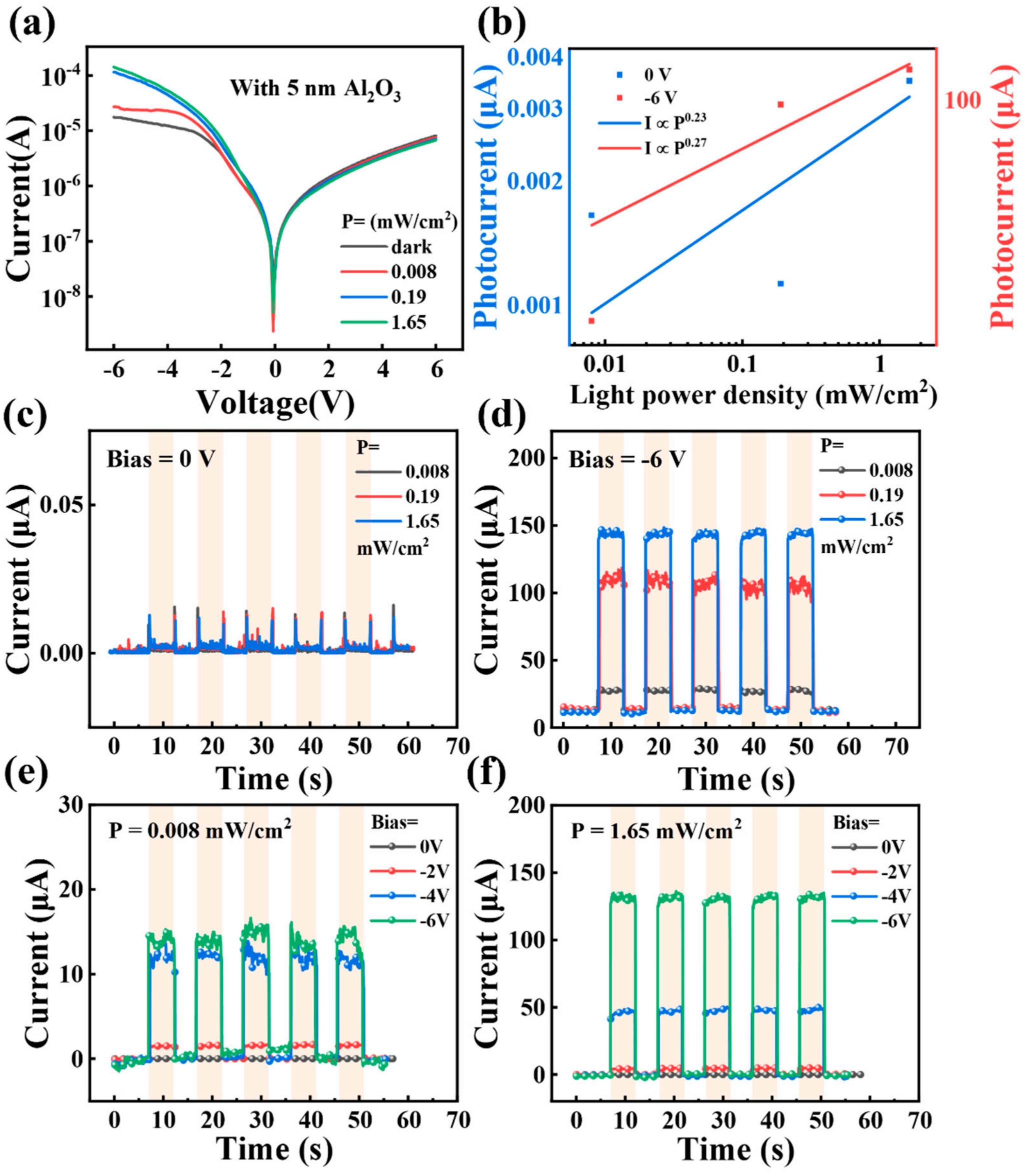


**Figure S4. (a) Relationship between photocurrent and bias voltage of GNR/$Al_2O_3$/Si photodetector with 5 nm $Al_2O_3$ at different optical power densities. (b) The fitting curve of the relationship between the photocurrent and optical power density measured under bias voltages of 0 V and −6 V. Dynamic current response of the photodetectors at different optical power densities with bias voltages of (c) 0 V and (d) -6 V. Dynamic current response of photodetectors at different bias voltages with optical power densities of (e) 0.008 mW/cm$^2$ and (f) 1.65 mW/cm$^2$.**

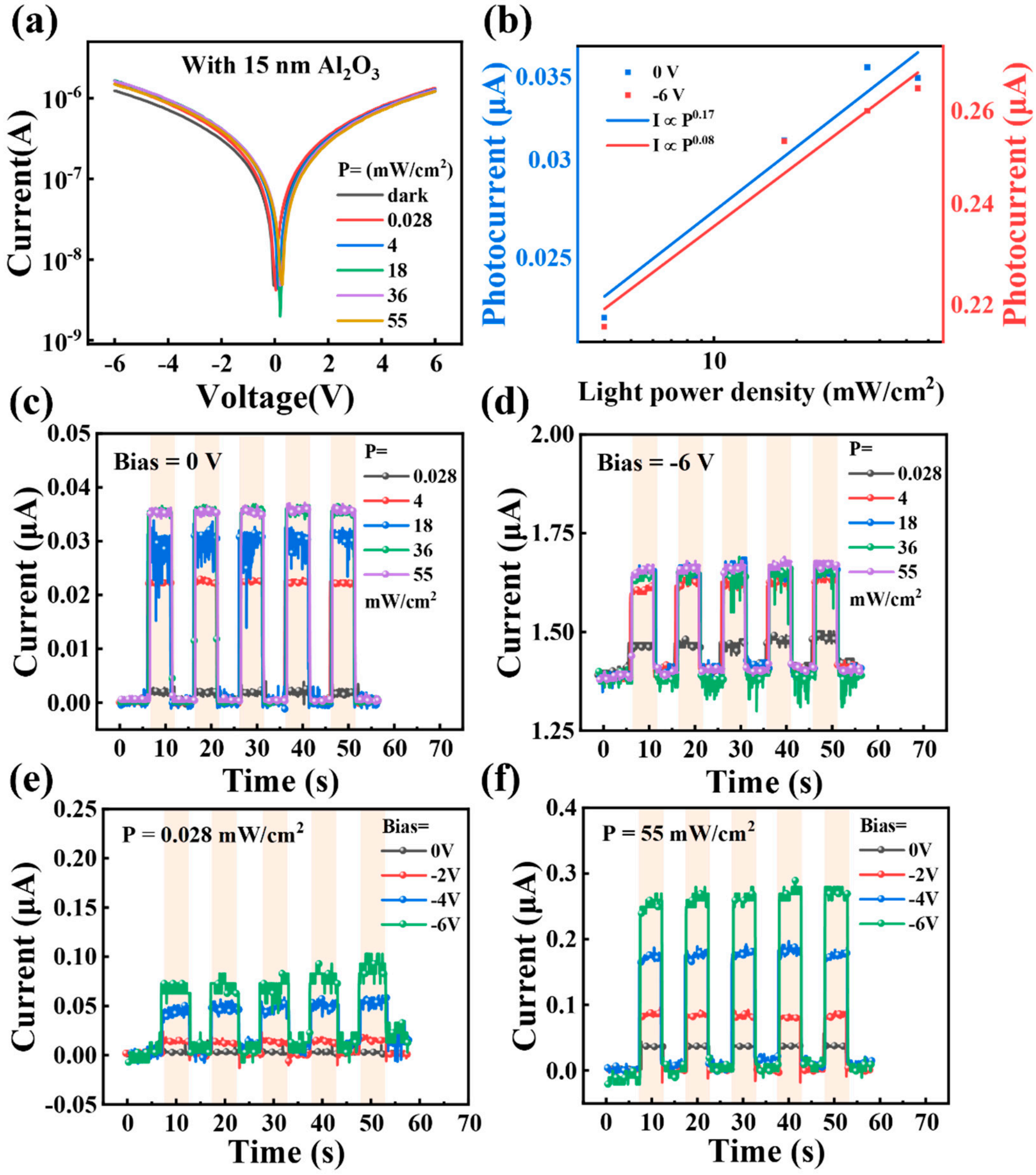


**Figure S5. (a) Relationship between photocurrent and bias voltage of GNR/$Al_2O_3$/Si photodetector with 15 nm $Al_2O_3$ at different optical power densities. (b) The fitting curve of the relationship between the photocurrent and optical power density measured under bias voltages of 0 V and −6 V. Dynamic current response of the photodetectors at different optical power densities with bias voltages of (c) 0 V and (d) -6 V. Dynamic current response of photodetectors at different bias voltages with optical power densities of (e) 0.028 mW/cm$^2$ and (f) 55 mW/cm$^2$.**

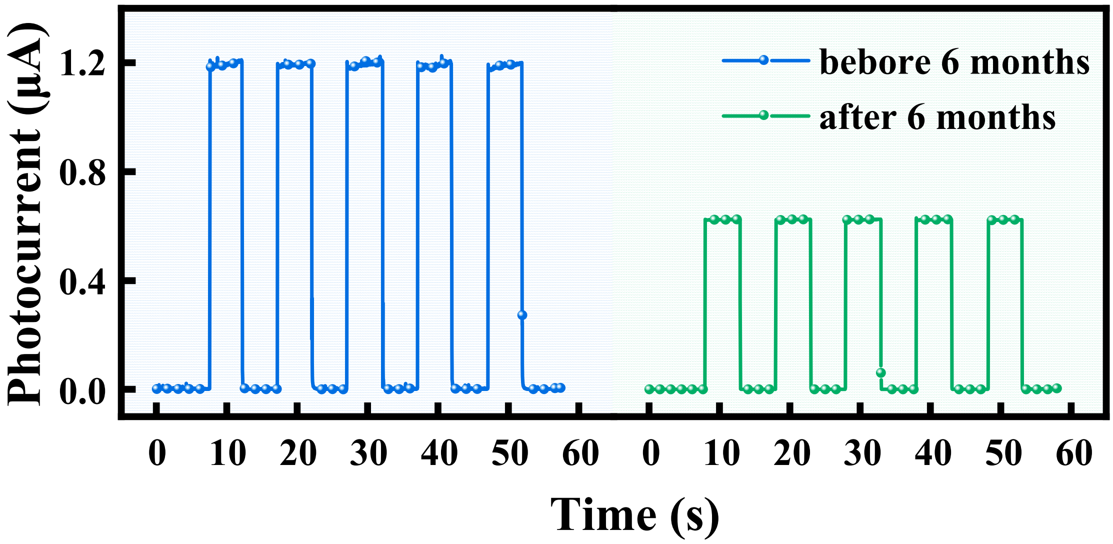


**Figure S6. the dynamic response curve of the GNR/$Al_2O_3$/Si photodetector after being placed for 6 months**

Table S1. Comparison of the performance of the photodetector in this paper with other graphene-related photodetectors

| Device Structure | Responsivity | Detectivity （Jones） | Response Time | Wavelength （nm） | Optical Power Density/Optical Power | References |
|---|---|---|---|---|---|---|
| **GNR/$Al_2O_3$/Si** | **159.55 A/W** | **$1.15\times10^{13}$** | **680/307.5μs** | **1064** | **0.038-39.2 $mW/cm^2$** | **This work** |
| Gr/Si | 39.5 mA/W | $10^{11}$ | 5/8 μs | 1500 | 0.14-0.73 $mW/cm^2$ | [1] |
| Gr/Si | 510 mA/W | | 130/135 μs | 532 | 0.06–5.7 $mW/cm^2$ | [2] |
| Gr/Si | 29 mA/W | $10^{11}$ | 93/110 μs | 850/940 | 5 $mW/cm^2$ | [3] |
| Gr/Si | 435 mA/W | $10^{9}$ | 1200/3000 μs | | | [4] |
| Gr p-n junction | 1.4-4.7 A/W | $10^{12}$ | 1.2/0.8 μs | 532/980/1550 | 15–27 $mW/cm^2$ | [5] |
| Gr/$SiO_2$/Si | 730 mA/W | $10^{13}$ | 320/750 μs | 410–950 | 0.5–30 $\mu W/cm^2$ | [6] |
| GNR/$Al_2O_3$/IGZO | 68 mA/W | $8.34\times10^{10}$ | 21/20 ms | 405–1550 | 0.22–0.78 $mW/cm^2$ | [7] |
| Gr/GaAs nanowire | 1.54 mA/W | | 71/194 μs | 532 | 2.05 mW | [8] |
| Gr/ZnO | 50 μA/W | | 32 ms | 365 | 2 $mW/cm^2$ | [9] |
| Gr/Ge | 1.27 A/W | $9.6\times10^{9}$ | | 1550 | 52.6 μW | [10] |
| $MoS_2$/Gr/GaAs | 19.9 mA/W | $4.86\times10^{10}$ | 46.8/557 μs | 808/1064 | 0.5 mW | [11] |
| Gr/$HfO_2$/a-$MoS_2$ | 5.36 A/W | | 68 μs | 473-2712 | 0.217 μW ~ 279 μW | [12] |
| Gr/Si/Gr oxide | 0.65 A/W | | 1 ms | 633 | 0 ~ 100 $mW/cm^2$ | [13] |
| GNR | 800 A/W | | 10 μs | 532/ 632 | 0.34 ~ 5 mW | [14] |
| Gr | 32 A/W | | 150 μs | 632 | 5 mW | [15] |
| GNR | 1.75 A/W | $>2\times10^{6}$ | | 632 /1470 /10000 | 2.5 $W/cm^2$ | [16] |
| UCNPs/Gr/GaAs | 5.97 mA/W | | | 980 | 19 $mW/cm^2$ | [17] |
| Gr/GaAs | 200 mA/W | | | 325/635 | 2 ~ 10 μW | [18] |
| Gr/$Al_2O_3$/GaAs | 5 mA/W | $2.88\times10^{11}$ | | 850 | 3 $mW/cm^2$ | [19] |
| Gr/InAs nanowire | 0.5 A/W | | | 1000 | 1 $W/cm^2$ | [20] |
| rGO/n-Si | 1.52 A/W | | 2/3.7 ms | 365-1200 | 0.036 ~ 5.4 $mW/cm^2$ | [21] |
| Ge nanodots/Gr | 44.5 mA/W | $2.16\times10^{10}$ | 18.7/18.7 μs | 1550 | 5 ~ 50 $mW/cm^2$ | [22] |
| Gr-Ge Schottky junction | 51.8 mA/W | $1.38\times10^{10}$ | 23/108 μs | 1550 | 0.14 ~ 18 $W/cm^2$ | [23] |
| all-carbon Gr-C60 hybrid nanostructure | 0.4 A/W | | | 10000 | 2.5 $W/cm^2$ | [24] |

# References


[1] Wang, C.; Dong, Y.; Lu, Z.; Chen, S.; Xu, K.; Ma, Y.; Xu, G.; Zhao, X.; Yu, Y. High Responsivity and High-Speed 1.55 μm Infrared Photodetector from Self-Powered Graphene/Si Heterojunction. *Sensors and Actuators A: Physical*. **2019**, *291*, 87-92.

[2] Periyanagounder, D.; Gnanasekar, P.; Varadhan, P.; He, J.-H.; Kulandaivel, J. High Performance, Self-Powered Photodetectors Based on a Graphene/Silicon Schottky Junction Diode. *Journal of Materials Chemistry C*. **2018**, *6*, 9545-9551.

[3] Lv, P.; Zhang, X.; Zhang, X.; Deng, W.; Jie, J. High-Sensitivity and Fast-Response Graphene/Crystalline Silicon Schottky Junction-Based Near-IR Photodetectors. *IEEE Electron Device Letters*. **2013**, *34*, 1337-1339.

[4] Novoselov, K. S.; Geim, A. K.; Morozov, S. V.; Jiang, D.; Katsnelson, M. I.; Grigorieva, I. V.; Dubonos, S. V.; Firsov, A. A. Two-Dimensional Gas of Massless Dirac Fermions in Graphene. *Nature*. **2005**, *438*, 197-200.

[5] Wang, G.; Zhang, M.; Chen, D.; Guo, Q.; Feng, X.; Niu, T.; Liu, X.; Li, A.; Lai, J.; Sun, D.; et al. Seamless Lateral Graphene P–N Junctions Formed by Selective in situ Doping for High-Performance Photodetectors. *Nature Communications*. **2018**, *9*.

[6] Li, X.; Zhu, M.; Du, M.; Lv, Z.; Zhang, L.; Li, Y.; Yang, Y.; Yang, T.; Li, X.; Wang, K.; et al. High Detectivity Graphene-Silicon Heterojunction Photodetector. *Small*. **2015**, *12*, 595-601.

[7] Ye, X.; Zheng, X.; Han, L.; Sun, Y.; Wang, L.; Li, Z.; Liu, W.; Liu, B.; Han, N.; Khan, S.; et al. High Performance Self-Powered Photodetectors Based on Graphene Nanoribbons/Al2O3/InGaZnO Heterojunctions. *IEEE Photonics Journal*. **2024**, *16*.

[8] Wu, Y.; Yan, X.; Zhang, X.; Ren, X. A Monolayer Graphene/GaAs Nanowire Array Schottky Junction Self-Powered Photodetector. *Applied Physics Letters*. **2016**, *109*.

[9] Chen, D.; Xin, Y.; Lu, B.; Pan, X.; Huang, J.; He, H.; Ye, Z. Self-Powered Ultraviolet Photovoltaic Photodetector Based on Graphene/ZnO Heterostructure. *Applied Surface Science*. **2020**, *529*.

[10] Kwon, M. G.; Kim, C.; Chang, K. E.; Yoo, T. J.; Kim, S.-Y.; Hwang, H. J.; Lee, S.; Lee, B. H. Performance Enhancement of Graphene/Ge Near-Infrared Photodetector by Modulating the Doping Level of Graphene. *APL Photonics*. **2022**, *7*.

[11] Qu, J.; Chen, J. Graphene/GaAs Schottky Junction Near-Infrared Photodetector With a MoS2 Quantum Dots Absorption Layer. *IEEE Transactions on Electron Devices*. **2022**, *69*, 4331-4336.
[12] Huang, Z.; Liu, J.; Zhang, T.; Jin, Y.; Wang, J.; Fan, S.; Li, Q. Interfacial Gated Graphene Photodetector with Broadband Response. *ACS Applied Materials & Interfaces*. **2021**, *13*, 22796-22805.
[13] Wang, Y.; Yang, S.; Lambada, D. R.; Shafique, S. A Graphene-Silicon Schottky Photodetector with Graphene Oxide Interlayer. *Sensors and Actuators A: Physical*. **2020**, *314*.
[14] Yu, J.; Zhong, J.; Kuang, X.; Zeng, C.; Cao, L.; Liu, Y.; Liu, Z. Dynamic Control of High-Range Photoresponsivity in a Graphene Nanoribbon Photodetector. *Nanoscale Research Letters*. **2020**, *15*.
[15] Liu, Y.; Xia, Q.; He, J.; Liu, Z. Direct Observation of High Photoresponsivity in Pure Graphene Photodetectors. *Nanoscale Research Letters*. **2017**, *12*.
[16] Yu, X.; Dong, Z.; Liu, Y.; Liu, T.; Tao, J.; Zeng, Y.; Yang, J. K. W.; Wang, Q. J. A High Performance, Visible to Mid-Infrared Photodetector Based on Graphene Nanoribbons Passivated with HfO2. *Nanoscale*. **2016**, *8*, 327-332.
[17] Wu, J.; Yang, Z.; Qiu, C.; Zhang, Y.; Wu, Z.; Yang, J.; Lu, Y.; Li, J.; Yang, D.; Hao, R.; et al. Enhanced Performance of a Graphene/GaAs Self-Driven Near-Infrared Photodetector with Upconversion Nanoparticles. *Nanoscale*. **2018**, *10*, 8023-8030.
[18] Li, X.; Lin, S.; Lin, X.; Xu, Z.; Wang, P.; Zhang, S.; Zhong, H.; Xu, W.; Wu, Z.; Fang, W. Graphene/h-BN/GaAs Sandwich Diode as Solar Cell and Photodetector. *Optics Express*. **2016**, *24*.
[19] Luo, L.-B.; Hu, H.; Wang, X.-H.; Lu, R.; Zou, Y.-F.; Yu, Y.-Q.; Liang, F.-X. A Graphene/GaAs Near-Infrared Photodetector Enabled by Interfacial Passivation with Fast Response and High Sensitivity. *Journal of Materials Chemistry C*. **2015**, *3*, 4723-4728.
[20] Miao, J.; Hu, W.; Guo, N.; Lu, Z.; Liu, X.; Liao, L.; Chen, P.; Jiang, T.; Wu, S.; Ho, J. C.; et al. High-Responsivity Graphene/InAs Nanowire Heterojunction Near-Infrared Photodetectors with Distinct Photocurrent On/Off Ratios. *Small*. **2015**, *11*, 936-942.
[21] Li, G.; Liu, L.; Wu, G.; Chen, W.; Qin, S.; Wang, Y.; Zhang, T. Self-Powered UV–Near Infrared Photodetector Based on Reduced Graphene Oxide/n-Si Vertical Heterojunction. *Small*. **2016**, *12*, 5019-5026.
[22] Gao, M.; Tian, Z.; Tang, S.; Han, X.; Zhang, M.; Xue, Z.; Zhu, W.; Mei, Y.; Chu, P. K.; Wang, G.; et al. Ambipolar Plasmon-Enhanced Photodetector

Built on Germanium Nanodots Array/Graphene Hybrid. *Advanced Materials Interfaces*. **2020**, *7*.
[23] Zeng, L.-H.; Wang, M.-Z.; Hu, H.; Nie, B.; Yu, Y.-Q.; Wu, C.-Y.; Wang, L.; Hu, J.-G.; Xie, C.; Liang, F.-X.; et al. Monolayer Graphene/Germanium Schottky Junction As High-Performance Self-Driven Infrared Light Photodetector. *ACS Applied Materials & Interfaces*. **2013**, *5*, 9362-9366.
[24] Yu, X.; Dong, Z.; Yang, J. K. W.; Wang, Q. J. Room-Temperature Mid-Infrared Photodetector in All-Carbon Graphene Nanoribbon-C60 Hybrid Nanostructure. *Optica*. **2016**, *3*.
